# Metabolic and hypoxic adaptation to anti-angiogenic therapy: a target for induced essentiality

**DOI:** 10.15252/emmm.201404271

**Published:** 2015-02-19

**Authors:** Alan McIntyre, Adrian L Harris

**Affiliations:** Hypoxia and angiogenesis Group, Department of Oncology, Weatherall Institute of Molecular Medicine, University of OxfordOxford, UK

**Keywords:** angiogenesis, anti-VEGF therapy, combination therapy, hypoxia, metabolism

## Abstract

Anti-angiogenic therapy has increased the progression-free survival of many cancer patients but has had little effect on overall survival, even in colon cancer (average 6–8 weeks) due to resistance. The current licensed targeted therapies all inhibit VEGF signalling (Table1). Many mechanisms of resistance to anti-VEGF therapy have been identified that enable cancers to bypass the angiogenic blockade. In addition, over the last decade, there has been increasing evidence for the role that the hypoxic and metabolic responses play in tumour adaptation to anti-angiogenic therapy. The hypoxic tumour response, through the transcription factor hypoxia-inducible factors (HIFs), induces major gene expression, metabolic and phenotypic changes, including increased invasion and metastasis. Pre-clinical studies combining anti-angiogenics with inhibitors of tumour hypoxic and metabolic adaptation have shown great promise, and combination clinical trials have been instigated. Understanding individual patient response and the response timing, given the opposing effects of vascular normalisation versus reduced perfusion seen with anti-angiogenics, provides a further hurdle in the paradigm of personalised therapeutic intervention. Additional approaches for targeting the hypoxic tumour microenvironment are being investigated in pre-clinical and clinical studies that have potential for producing synthetic lethality in combination with anti-angiogenic therapy as a future therapeutic strategy.

## Introduction

The original version of the ‘Hallmarks of Cancer’ highlighted the role of neo-angiogenesis in tumour progression (Hanahan & Weinberg, 2000). Targeting angiogenesis as a tumour therapy was first hypothesised over four decades ago (Folkman, 1971). In the updated ‘Hallmarks of Cancer: The Next Generation,’ the ‘deregulation of cellular energetics’ has also been added (Hanahan & Weinberg, 2011). This was linked to the renewed interest and substantial findings of the role of metabolic reprogramming in tumour progression. Low oxygen level (hypoxia) was overlooked in both versions of the ‘Hallmarks of Cancer’ but is inextricably linked with nearly all the hallmarks (Kroemer & Pouyssegur, 2008). In particular, the regulation of both angiogenesis and metabolism by hypoxia is well documented (Semenza, 2014). Here, we review the link between these three important aspects of tumour physiology with a particular focus on combination therapeutic approaches.

## Tumour angiogenesis

**Figure 1:**
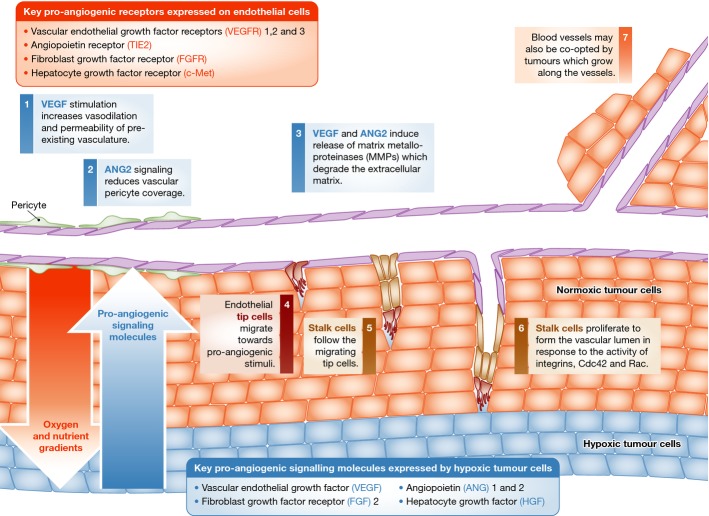
Angiogenesis into the hypoxic tumour microenvironment This figure highlights the role of hypoxia-regulated proteins in the angiogenic process.

As tumours grow, they require additional nutrients and oxygen, and induce new blood vessels. The growth of new blood vessels (angiogenesis) is a process tightly regulated by a balance of pro- and anti-angiogenic signalling in normal physiology. In tumour tissues, this balance is lost resulting in a vasculature with a chaotic, leaky architecture. Angiogenesis is a multistage process regulated by numerous growth factors and their receptors (Fig1). Key growth factors (and their cognate receptors on endothelial cells shown in brackets) in the angiogenic process include VEGF, VEGFR1, 2 and 3, ANG1 and 2 (TIE2), FGF2 (FGFR1, 2, 3, 4) and HGF (c-Met) (Welti *et al*, 2013). These cross talk with additional signalling pathways, in particular the Notch pathway, which is required for patterning of functional vasculature (Blanco & Gerhardt, 2013; Welti *et al*, 2013). Vascular endothelial growth factor (VEGF), one of the main drivers of angiogenesis, increases vasodilation and permeability of pre-existing vasculature (Welti *et al*, 2013). TIE2 and angiopoietin 2 (ANG2) signalling reduce vascular pericyte coverage (Augustin *et al*, 2009). These induce the release of proteases including matrix metalloproteinases, resulting in degradation of the extracellular matrix (Bridges & Harris, 2011). Endothelial tip cells migrate towards the pro-angiogenic stimuli, and are followed by stalk cells, which proliferate to form the vascular lumen, in response to activity of integrins, Cdc42 and Rac (Bridges & Harris, 2011; Welti *et al*, 2013). Blood vessels may also be co-opted by tumours. Tumour vessel co-option (Fig1) is the utilisation of pre-existing vasculature by growing or migrating along these vessels. It has mostly been identified in well-vascularised tissues of the lung, liver and brain (Donnem *et al*, 2013; Pezzella & Harris, 2014). Metabolically, vessel-co-opted tumours have higher expression of genes encoding mitochondrial proteins, suggesting a greater use of TCA cycle (Donnem *et al*, 2013). Distinguishing co-opted vessels from neo-angiogenic vessels is difficult and a mixed phenotype of vascularisation is often identified (Donnem *et al*, 2013), suggesting combined approaches for targeting both types of vasculature may be required for some tumours (Pezzella & Harris, 2014). Many of the factors that regulate angiogenesis are increased in the hypoxic conditions found in tumours (Semenza, 2014). However, tumour anti-angiogenic anti-VEGF therapy increases hypoxia (Franco *et al*, 2006; De Bock *et al*, 2011).

## Hypoxia

Hypoxia arises from the combination of high metabolic and proliferative rates, that is consumption and aberrant tumour vascularisation with poor oxygen delivery (Semenza, 2014). Clinically, tumour hypoxia is associated with poor patient prognosis and resistance to chemotherapy (Rebucci & Michiels, 2013) and radiotherapy (Multhoff *et al*, 2014) and poor outcome as an independent factor, regardless of treatment modality. Hypoxia regulates the expression of genes under the transcriptional control of hypoxia-inducible factor-1α and hypoxia-inducible factor-2α (HIF1α and HIF2α), which heterodimerise with HIF1β and bind the hypoxia response element (HRE) in the promoter of many genes (Choudhry *et al*, 2014). HIF1α and HIF2α mRNAs are constitutively expressed, but the proteins produced are constantly targeted for degradation in normoxic conditions by prolyl-hydroxylases, which require O_2_ as a cofactor (Shen & Kaelin, 2013). Upon hydroxylation, HIF1α and HIF2α interact with VHL, which mediates HIF ubiquitination and degradation. Inactivating mutations in VHL lead to HIF stabilisation in normoxia and are often found in renal cell carcinoma (RCC) (Shen & Kaelin, 2013). Growth factor signalling cascades PI3K/AKT/mTOR and MAPK also regulate the expression and phosphorylation of HIF1α and HIF2α (Agani & Jiang, 2013). Many HIF-regulated genes trigger more aggressive growth and survival, and contribute to the major hallmarks of cancer. HIF regulates genes in key processes of tumour growth in particular metabolism and angiogenesis (Favaro *et al*, 2011; Semenza, 2014). Metabolic genes increased by HIF1 in response to hypoxia include many of those involved in glycolysis: GLUT1, GLUT3, PDK1, PKM2, PFKFB3, GYS1, ENO1, LDHA, HK2 and GAPDH (Favaro *et al*, 2011). Angiogenesis genes increased by HIF in response to hypoxia include VEGF, FLT-1, ANG1, 2, TIE2, PDGF, MMP2, 9 and FLK1 (Favaro *et al*, 2011; Semenza, 2014) (Fig1).

Hypoxia increases invasion and metastasis, and HIF1 regulates the expression of key genes (c-Met, CXCR4, RIOK3 and LOX), of these processes (De Bock *et al*, 2011; Singleton *et al*, 2014). Hypoxia also increases autophagy, promoting survival of hypoxic tumour cells (Brahimi-Horn *et al*, 2011). High numbers of tumour-associated macrophages (TAMs) accumulate in hypoxic areas of tumours (Gilkes *et al*, 2014). TAMs are attracted to the hypoxic microenvironment by increased expression of monocytic chemotactic proteins, VEGF, semaphorin 3A and interleukin 1 (Gilkes *et al*, 2014).

The hypoxic tumour extracellular microenvironment is also acidic because of increased production of metabolic acids, CO_2_ and lactic acid (from glycolysis) (Parks *et al*, 2013) and longer diffusion distances to functional blood capillaries. The optimal range of intracellular pH (pH_i_) is narrow, such that only a fraction of a pH_i_-unit change can lead to aberrant function or even death. Regulation of pH_i_ is required to maintain optimum conditions for signalling such as mTOR (Balgi *et al*, 2011). Many pH regulatory proteins have increased expression and/or activity in hypoxia including monocarboxylate transporters 1 and 4 (MCT1, 4) that export lactate, and carbonic anhydrase 9 (CA9) (Parks *et al*, 2013) that hydrates extracellular CO_2_ to produce a steeper efflux gradient (Swietach *et al*, 2014).

## Tumour metabolism

The metabolic profiles of tumour cells are dramatically different from normal adult cells as their metabolic requirements shift based on phenotypic changes, including increased proliferation and survival in the acidic- and nutrient-depleted tumour microenvironment. Energy generation, anabolism and maintenance of redox potential are promoted in normoxia and hypoxia as the need for biomass precursors (nucleotides, lipids and proteins) and ATP increase (Schulze & Harris, 2012). Tumour metabolism is regulated by a number of different factors: most notably HIF (hypoxia), AMP-activated protein kinase (AMPK) (nutrient sensing), growth factor signalling and oncogenes (RAS, MYC, etc.) (Schulze & Harris, 2012). AMPK is activated in response to increased AMP/ATP ratios and hypoxia. It induces a metabolic shift away from glycolysis to oxidative phosphorylation and autophagy (Liang & Mills, 2013).

**Figure 2:**
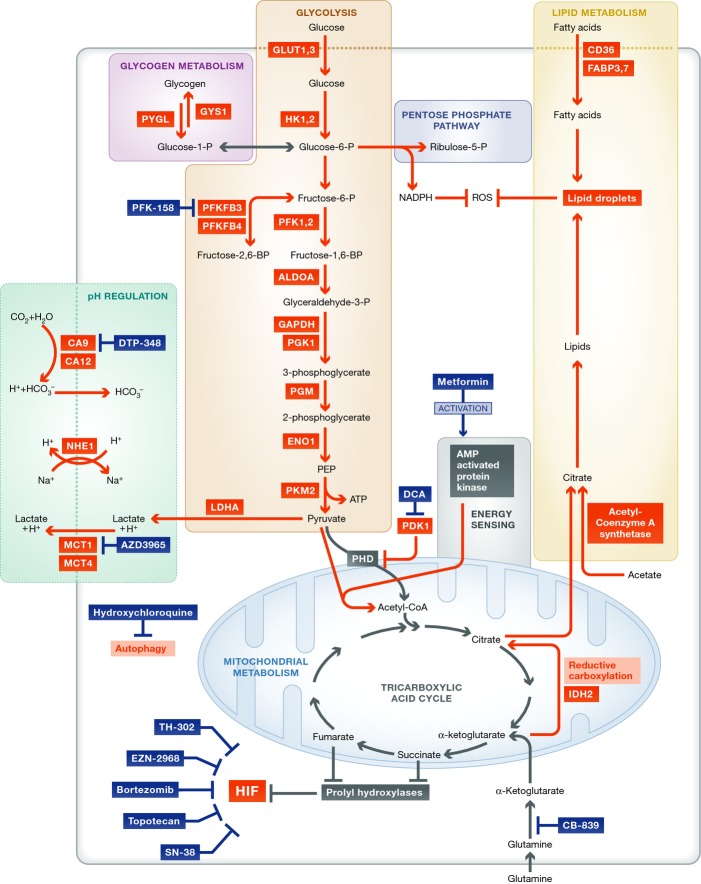
Metabolic reprogramming in the hypoxic microenvironment This figure shows the metabolic processes that are upregulated in response to hypoxia and the therapeutic drugs that target these processes, which are in clinical trials (highlighted in white boxes). Proteins in red have increased expression or activity in hypoxia. Arrows in red denote increased flux in hypoxia. ALDOA, aldolase A; CA, carbonic anhydrase; CD36, fatty acid translocase; DCA, dichloroacetate; ENO1, enolase 1; FABP, fatty acid binding protein; GAPDH, glyceraldehyde 3-phosphate dehydrogenase; GLUT, glucose transporter; GYS1, glycogen synthase; HK, hexokinase; HIF, hypoxia-inducible factor; IDH2, isocitrate dehydrogenase 2; LDHA, lactate dehydrogenase A; MCT, monocarboxylate transporter; NHE1, sodium hydrogen antiporter 1; PDH, pyruvate dehydrogenase; PDK1, pyruvate dehydrogenase kinase 1; PFK, phosphofructokinase; PFKFBP, phosphofructokinase bisphosphatase; PGK1, phosphoglycerate kinase 1; PGM, phosphoglycerate mutase; PKM2, pyruvate kinase M2; PYGL, liver glycogen phosphorylase.

Here, we focus on the metabolism of hypoxic cells (Fig2). Hypoxia reduces oxidative phosphorylation by preventing pyruvate from entering the tricarboxylic acid (TCA) cycle, via up-regulation of pyruvate dehydrogenase kinase (PDK1) and then inhibition of pyruvate dehydrogenase (PDH). Pyruvate is instead broken down to lactic acid and extruded via MCT4 (Brahimi-Horn *et al*, 2011). However, mitochondria remain active in the hypoxic microenvironment (Sun & Denko, 2014). Hypoxia increases glutamine uptake and metabolism. Glutamine-derived α-ketoglutarate is used to replenish intermediates of the tricarboxylic acid (TCA) cycle as an alternative to pyruvate. This includes citrate produced via reductive carboxylation of α-ketoglutarate, a process promoted by hypoxia. The reductive carboxylation-derived citrate can be used in lipid synthesis, which is required for tumour growth (Sun & Denko, 2014).

The pentose phosphate pathway (PPP) is up-regulated in cancer, and stabilisation of HIF1α increases expression of genes involved in the PPP (Riganti *et al*, 2012). The PPP generates nucleotides and also NADPH, an important reducing agent required for lipid, nucleotide and amino acid synthesis, and, in particular, glutathione and ROS protection (Favaro *et al*, 2012). Hypoxia increases expression of glycogen synthase 1 (GYS1) in a HIF-dependent manner, which increases glycogen stores. HIF-dependent increased expression of PYGL breaks down the glycogen stores, and knock-down of this enzyme was shown to reduce entry of glucose into the PPP and induce ROS-dependent senescence (Favaro *et al*, 2012), highlighting the importance of glycogen metabolism in protection from free radicals (Favaro *et al*, 2012). Similarly, hypoxia-regulated 6-phosphofructo-2-kinase/fructose-2,6-biphosphatase 4 (PFKFB4) is essential for regulating entry to glycolysis or PPP for NADPH production (Ros & Schulze, 2013). Hypoxia-inducible PFKFB3 conversely increases glycolytic flux due to its greater kinase activity (Ros & Schulze, 2013).

Hypoxia and RAS transformation increase scavenging of serum fatty acids in particular lysophospholipids, and hypoxia reduces *de novo* lipogenesis (Kamphorst *et al*, 2013). Furthermore, fatty acid uptake and storage is increased in hypoxia via fatty acid binding proteins 3 (FABP3) and 7 (FABP7) (Bensaad *et al*, 2014). An increase in fatty acid storage in hypoxia protects against ROS toxicity (Bensaad *et al*, 2014).

Further to those already discussed, additional metabolic proteins have increased expression or activity in hypoxia including HK1, PFK1, PFK2, ALDOA, PGK1, PGM (glycolysis), NHE1 (pH regulation), CD36 and acetyl-coenzyme A synthetase (lipid metabolism) (Bensaad & Harris, 2014).

In addition to HIF and hypoxia-regulating metabolic processes, metabolic intermediates (including succinate and fumarate) can increase HIF stabilisation via inhibition of prolyl-hydroxylases (Schulze & Harris, 2012). This highlights the bidirectional regulation of hypoxia/HIF and metabolism as is also found with angiogenesis.

A likely confounding factor of targeting metabolic processes alone in tumours is the plasticity of metabolic networks. To complicate this further, the identified metabolic interactions with stromal cells (Martinez-Outschoorn *et al*, 2014) make our current understanding of tumour metabolism, which is mostly derived from 2D culture experiments, less conclusive with regard to intervention. In addition, metabolic reprogramming of endothelial cells plays an important role in the process of angiogenesis (Goveia *et al*, 2014).

## Anti-angiogenic therapies

Most anti-angiogenic therapies target the key pro-angiogenic factors or their endothelial cell-expressed receptors including VEGF, VEGFR1, 2 and 3, ANG 1 and 2, TIE2, FGF2, FGFR, c-Met and HGF (Bridges & Harris, 2011). The first anti-angiogenic therapy Avastin (bevacizumab) was approved by the Food and Drug Administration (FDA) in 2003. This inhibits the main driver of angiogenesis, VEGFA. A list of FDA-approved anti-angiogenic therapies is shown in Table1. The receptor tyrosine kinase (RTK)-based inhibitor approaches to anti-angiogenic therapy are likely also effecting tumour RTK signalling cascades, which regulate tumour proliferation, survival, metabolism and HIF (Shimobayashi & Hall, 2014).

**Table 1:** A list of all FDA-approved antiangiogenic therapies (2014).

Compound	Target	Indication
Antibody-based therapies		
Bevacizumab (Avastin)	Anti-VEGF antibody	Glioblastoma, metastatic colorectal cancer, metastatic RCC, some non-small cell lung cancers
Aflibercept (Eylea)	VEGF-trap recombinant fusion protein of VEGF-binding domains from VEGFR	Metastatic colorectal cancer
Ramucirumab (Cyrazma)	Human monoclonal VEGFR2 antibody inhibits VEGF binding	Advanced gastric or gastro-oesophageal junction adenocarcinoma
Small molecular inhibitors		
Axitinib (Inlyta)	VEGFR1-3, PDGFRβ, and c-KIT	Advanced RCC
Cabozantinib (Cometriq)	VEGFR1-3, MET	Metastatic medullary thyroid cancer
Everolimus (Afinitor)	mTOR	RCC, neuroendocrine tumours
Pazopanib (Votrient)	VEGFR1-3, PDGFR, c-KIT	RCC
Regorafanib (Stivarga)	VEGFR1-3, PDGFRβ, TIE2	Metastatic colorectal cancer
Sorafenib (Nexavar)	VEGFR1-3, PDGFR, RAF	Hepatocellular carcinoma, RCC
Sunitinib (Sutent)	VEGFR1-3, PDGFR, c-KIT, FLT3, RET, CSF-1R	RCC, neuroendocrine tumours
Vendatanib (Caprelsa)	VEGFR1-3, EGFR, RET	Medullary thyroid cancer in patients with unrespectable locally advanced or metastatic disease

RCC, renal cell carcinoma.

In addition to the anti-angiogenic therapies described above, there is an additional class of inhibitors, the vascular disrupting agents (VDA) such as Zybrestat (combretastatin A-4 phosphate; CA4P) (Nathan *et al*, 2012). These work by selectively targeting immature, rapidly proliferating endothelial cells, inducing apoptosis or disrupting endothelial cytoskeleton resulting in vascular collapse (Nathan *et al*, 2012). CA4P has also been combined with bevacizumab in pre-clinical and clinical studies but had substantial toxicity (Nathan *et al*, 2012).

Three main vascular responses to anti-angiogenic therapy have been described clinically utilising MRI. These are reduced perfusion, no perfusion response and increased perfusion also referred to as vascular normalisation (Mehta *et al*, 2011; Van der Veldt *et al*, 2012; Batchelor *et al*, 2013). Many studies have identified reduced tumour perfusion as an early and most frequent response to anti-angiogenic therapy, these include studies where no samples with vascular normalisation were identified (Willett *et al*, 2004; Mehta *et al*, 2011; Van der Veldt *et al*, 2012). Consistent with the identified reduced perfusion, studies have shown robust and significant increases in hypoxia in approximately half the patients or more (Hattingen *et al*, 2011; Mehta *et al*, 2011; Yopp *et al*, 2011; DeLay *et al*, 2012). These include increased expression of a hypoxic gene signature in 10 of 21 patients (Mehta *et al*, 2011), increased immunohistochemical staining for CA9 and HIF averaging a threefold increase in 9 of 21 tumours (DeLay *et al*, 2012) and increased, relative tumour hypoxia as analysed by high-resolution T2 and T2′ mapping (Hattingen *et al*, 2011). Bevacizumab-induced reductions in tumour perfusion were significantly associated with increased HIF1α or CA9 expression in primary liver cancer (Yopp *et al*, 2011).

A few studies have identified vascular normalisation mainly at the start of therapy, in half the assessed tumours or less, but not in other tumours, which showed reduced perfusion or no perfusion response (Sorensen *et al*, 2012; Batchelor *et al*, 2013). Increased survival effects were identified in patients who responded to anti-angiogenic therapy with increased tumour perfusion, due to increased chemotherapeutic delivery (Batchelor *et al*, 2013).

Additional clinical studies directly examining the effects of anti-angiogenics on perfusion, hypoxia and metabolic profiles should be pursued to enable more meaningful conclusions regarding the frequency of the different responses across tumour types and anti-angiogenic therapies. However, given the opposing responses to anti-angiogenic therapy, it is clear that clinically useable biomarkers of individual longitudinal response to anti-angiogenics need to be developed and included as part of clinical practice with anti-angiogenics. This is required to personalise combination approaches, for example, to include radiotherapy and chemotherapy for the window of vascular normalisation if present and targeting of hypoxic/metabolic adaptation once reduced perfusion is identified.

Anti-angiogenic therapies increase progression-free survival in many tumour types and overall survival in a few tumours, but this is variable. For example, a phase III clinical trial in metastatic colorectal cancer identified an increase in progression-free survival from 6.2 to 10.6 months when patients were treated with bevacizumab in combination with irinotecan, fluorouracil and leucovorin. An increase in overall survival was also identified (10.6 versus 15.6 months) (Hurwitz *et al*, 2004; Mittal *et al*, 2014). Similar results are seen in other cancer types with increases in progression-free survival in most studies and increases in overall survival in some studies with bevacizumab and additional agents (Mittal *et al*, 2014).

What is intriguing is that there was no effect in most adjuvant studies, suggesting the paradigm of dormancy until VEGF is activated and there is an angiogenic switch, needs revising (Maru *et al*, 2013). A few studies have shown improved disease-free survival whilst on treatment, an effect that is lost or reversed once anti-angiogenic treatment is stopped (Mountzios *et al*, 2014). However two studies with bevacizumab in ovarian cancer showed increases of disease-free survival which were much shorter than the duration of treatment (Mountzios *et al*, 2014) suggesting an optimal duration rather than unceasing treatment may be the best fit, this is complicated by individual vascular and tumour responses. However, most studies show that continuing with anti-angiogenic therapy is of benefit to progression-free survival. This again highlights the requirement for incorporation of a robust biomarker of response for anti-angiogenic therapy.

Although increased metastasis with anti-angiogenic therapy was found in pre-clinical models, extensive reviews of patterns of relapse on and off bevacizumab do not support this pre-clinical observation (Welti *et al*, 2013). One possible explanation for this is the effect of additional chemotherapeutic drugs combined with anti-angiogenics in the clinic such as doxorubicin, topotecan and gemcitabine, which counteract the sunitinib-induced metastatic dissemination of the Lewis lung carcinoma xenograft models (Rovida *et al*, 2013).

To explain the lack of overall patient survival, many mechanisms of resistance to anti-angiogenic therapy have been identified which can explain vascularisation despite treatment (Bridges & Harris, 2011). One can propose two main types of resistance to anti-angiogenic therapy, which are intrinsic resistance and adaptive resistance (Bergers & Hanahan, 2008).

Intrinsic resistance is due to underlying molecular differences in the tumour and its vasculature. Potential causes of intrinsic resistance include the following: differential expression of the VEGF pro- and anti-angiogenic isoforms, VEGF and VEGFR polymorphisms, limited penetration of antibodies into the tumour mass and pre-existing tumour microenvironment which results in a lack of effect such as dependence on vessel co-option, vessels that are well differentiated, or stimulated by pathways other than VEGF will also be resistant (Bergers & Hanahan, 2008; Donnem *et al*, 2013; Welti *et al*, 2013; Stapor *et al*, 2014). Notch signalling can down-regulate VEGF signalling and induce resistance (Li *et al*, 2011).

Adaptive resistance is due to changes that occur as a result of the effects of the therapy on the tumour and its vasculature. Causes of adaptive resistance may include the following: tumour expression and metabolic reprogramming by HIF after induction of hypoxia, hypoxia-induced increased invasion or vessel co-option, up-regulation of alternative pro-angiogenic factors by the tumour or by the stromal cells such as TAM recruited by hypoxia, and increased recruitment of protective pericyte coverage may also occur (Bergers & Hanahan, 2008; Welti *et al*, 2013; Gilkes *et al*, 2014).

An additional factor confounding anti-angiogenic therapy is the identification of six distinct types of vessels that coexist within tumours. These are mother vessels, capillaries, glomeruloid microvascular proliferations and vascular malformations which develop by angiogenesis from pre-existing vessels and feeder arteries and draining veins which develop by arterio-venogenesis (Nagy *et al*, 2010). Some of these do not require VEGF-A for maintenance, making VEGF-targeting strategies redundant (Sitohy *et al*, 2012). Further to the resistance mechanisms that explain the lack of increase in overall patient survival, a number of clinical factors could reduce the quantifiable effects of anti-angiogenics, including the lack of patient enrichment with predictive biomarkers, patient crossover in clinical trials and subsequent therapies post progression.

## Tumour response to anti-angiogenic therapy

Tumour microenvironmental and metabolic response to anti-angiogenic therapy can play an important role in resistance. Successful therapy against VEGF induces cell death and hypoxia because of reduction in vessel perfusion and will therefore activate gene transcription programmes that respond to hypoxia. These include HIF1 and HIF2, the unfolded protein response (UPR) (Rouschop *et al*, 2013) and ATF4 (Pike *et al*, 2012). Thus, in contrast to long-term development of cytotoxic drug resistance, hypoxia can induce resistance as part of the normal physiological response to reduced perfusion rapidly. This may partly explain the few weeks of overall survival or progression-free survival generally achieved.

Approaches to understanding tumour adaptation to anti-angiogenic therapy have utilised expression arrays of treated patient samples (DeLay *et al*, 2012), xenografts (Kumar *et al*, 2013; Gokmen-Polar *et al*, 2014; Sounni *et al*, 2014), post-treatment xenografts (Sounni *et al*, 2014) and xenograft models of resistance (Jahangiri *et al*, 2013). Additional investigations have utilised magnetic resonance spectrography (MRS) to examine the tumour metabolic responses to anti-angiogenic therapy in xenografts (Keunen *et al*, 2011) and clinical studies (Hattingen *et al*, 2011).

An increase in hypoxia and the hypoxia-regulated genes CA9 and c-Met was identified in bevacizumab-resistant glioblastomas after treatment (Jahangiri *et al*, 2013). Expression analysis of a bevacizumab-resistant glioblastoma cell line revealed that changes in genes regulating energy metabolism were predominant and that HIF was the key driver of identified changes. Genes regulating glycolysis and the PPP were increased, whereas genes regulating oxidative phosphorylation were decreased (Kumar *et al*, 2013). Transcription factors that regulate UPR in response to endoplasmic reticulum (ER) stress were increased in response to bevacizumab treatment (Kumar *et al*, 2013). Bevacizumab treatment of a glioblastoma cell line increased expression of HIF-regulated FABP3 and 7, which were responsible for lipid accumulation in hypoxia (Bensaad *et al*, 2014). A recent study investigated metabolic, proteomic and transcriptomic changes in cancer xenografts after withdrawal of anti-angiogenic therapy (sunitinib and sorafenib). Post-treatment xenograft metabolism shifted away from glycolysis and towards TCA and lipid metabolism following an initial decrease in genes regulating fatty acid metabolism during anti-angiogenic therapy (Kumar *et al*, 2013; Sounni *et al*, 2014).

MRS analysis of bevacizumab-treated tumours showed increased accumulation of metabolites previously associated with brain tumour hypoxia. These included increased lactate, alanine, choline and mobile lipids (Keunen *et al*, 2011). Bevacizumab increased hypoxia and impaired oxidative metabolism in a ^31^P/^1^H MRSI and MRI study of 16 glioblastoma patients (Hattingen *et al*, 2011).

## Induced essentiality

The above term reflects a variation on the term synthetic lethality (Ashworth *et al*, 2011). The implication is that a treatment-induced pathway then becomes essential for tumour survival. Considering that most tumours already have areas of hypoxia, anti-VEGF therapy will induce even greater dependence on hypoxia adaptations. Thus, the response to bevacizumab is dependent upon tumour susceptibility to hypoxia-induced apoptosis (Selvakumaran *et al*, 2008). As pre-clinical and clinical data have shown an increase in tumour hypoxia, and a metabolic adaptation driven by HIF in response to anti-angiogenic therapy, it is likely that targeting vascularisation and hypoxic adaptation in combination will yield improved therapeutic results and may be able to substantially prolong the duration of effect. However, additional unbiased functional screening of survival mechanisms in hypoxia may also provide interesting, previously unrecognised, hits. Approaches targeting HIF-driven changes in combination with anti-angiogenic therapy have shown promising results in pre-clinical models.

Dichloroacetate (DCA), a PDK inhibitor, increases oxidative phosphorylation. DCA enhanced bevacizumab treatment of glioblastoma cell lines (Kumar *et al*, 2013) and overcame sorafenib resistance in hepatocellular carcinoma xenografts (Shen *et al*, 2013). DCA treatment reduced lactate, increased ROS and ATP levels and significantly potentiated apoptosis in combination with sorafenib (Shen *et al*, 2013). Knock-down or inhibition of CA9, a key pH regulatory enzyme, enhanced bevacizumab therapy of colon and glioblastoma xenografts (McIntyre *et al*, 2012).

Knock-down of HIF1α reduced growth in combination with anti-angiogenic therapy in neuroblastoma xenografts (Hartwich *et al*, 2013). Similarly, combining HIF1 inhibition, by the topoisomerase inhibitor topotecan, with bevacizumab increased their anti-tumour activity synergistically in neuroblastoma xenografts (Hartwich *et al*, 2013).

Knock-down or inhibition of c-Met in bevacizumab-resistant glioblastoma xenografts increased sensitivity to bevacizumab and reduced invasion and survival in hypoxia (Jahangiri *et al*, 2013). Inhibition of autophagy enhanced the anti-cancer effect of bevacizumab in hepatocellular carcinoma (Guo *et al*, 2013).

Adaptation to anti-angiogenic therapy (sunitinib) withdrawal increases metastatic dissemination and xenograft regrowth (Sounni *et al*, 2014). This could be reduced with fatty acid synthase (FASN) inhibition (orlistat) or knock-down after sunitinib withdrawal (Sounni *et al*, 2014).

Other targets in tumour metabolism and hypoxia have been investigated, but not yet in combination with anti-angiogenic therapy. These offer additional options for future combination investigations. One study utilised macrophages, which migrate to the hypoxic area of tumours, to target a hypoxia-induced therapeutic adenovirus to prostate cancers (Muthana *et al*, 2011). The use of HRE promoter sequences to target induction of gene therapy approaches in the hypoxic microenvironment is a possibility, but has major issues for delivery.

Many strategies to target HIF directly have been developed. These include the use of SN-38, a camptothecin analogue that reduces transcriptional induction of HIF1α (Jeong *et al*, 2014b), and PX-478, which reduced HIF1α protein levels and trans-activating capacity (Lee & Kim, 2011). A recent study identified a cyclic peptide inhibitor that specifically inhibits HIF1α binding to HIF1β, preventing HIF1α trans-activating capacity (Miranda *et al*, 2013).

*In vitro* investigations of additional metabolic enzyme inhibitors have shown an effect on reducing cell viability. An inhibitor of a HIF target gene, glucose transporter GLUT1 (STF-31), was identified in a screen to target VHL-deficient RCC (Chan *et al*, 2011). MCT1 inhibition by AZD3965 reduced xenograft growth rate and increased sensitivity to radiotherapy (Bola *et al*, 2014).

It is likely that metabolic inhibitors will only work on the subset of cancers for which these pathways are critical, but inducing hypoxia may make them more essential.

## Clinical trials

There are few clinical trials combining inhibitors of hypoxia or metabolism with anti-angiogenics. We identified 11 on the clinical trials database ClinicalTrials.gov (Table2). NCT000520533 combined cG250, a non-inhibiting CAIX antibody, and sunitinib in advanced RCC; however, this study was terminated due to toxicity. NCT01578551 combines metformin with bevacizumab in advanced/metastatic pulmonary adenocarcinoma. An additional clinical trial is investigating the combination of metformin and everolimus in recurrent or progressive endometrial cancer (NCT01797523). Bevacizumab has also been combined with the c-Met inhibitor tivantinib (NCT10749384).

**Table 2:** A list of clinical trails which combine targeting of HIF, HIF target genes, metabolism or hypoxia with antiangiogenic therapy.

Clinical trials identifier	Phase	Treatment	Tumour type and setting	Response
NCT00520533		cG250 and sunitinib	Advanced RCC	Study terminated due to toxicity
NCT01578551	II	Metformin plus paclitaxel/carboplatin/bevacizumab	Previously untreated advanced/metastatic pulmonary adenocarcinoma	Ongoing
NCT01797523	II	Metformin plus everolimus and letroxole	Recurrent or progressive endometrial cancer	Ongoing
NCT01749384	I	Bevacizumab and tivantinib (c-MET inhibitor)	Solid tumours that are metastatic or cannot be removed by surgery	Ongoing
NCT01497444	I/II	Sorafenib and TH-302	Advanced kidney cancer or liver cancer	Ongoing
NCT01381822	I	TH-302 in combination with sunitinib	Advanced RCC, GISTs and pancreatic neuroendocrine tumours	Ongoing
NCT01403610	II	Bevacizumab followed by TH-302	Recurrent high grade astrocytoma	Ongoing
NCT01485042	I	Pazopanib plus TH-302	Advanced solid tumours	Ongoing
NCT00548418	II	Bevacizumab and topotecan with cisplatin	Recurrent/persistent cervical cancer	Ongoing
NCT00671112	I	Everolimus and bortezomib	Relapsed or refractory lymphoma	Ongoing
NCT02142803	I	Bevacizumab and MLN0128 (mTOR inhibitor)	Recurrent glioblastoma or advanced solid tumours	Ongoing

RCC, renal cell carcinoma; GISTs, gastrointestinal stromal tumours.

There are four phase I/II clinical trials combining the hypoxia-activated prodrug TH-302, an alkylating agent, (Chawla *et al*, 2014) with an anti-angiogenic therapy in a variety of tumours (NCT01497444, NCT01381822, NCT01403610, NCT01485042). There is an additional study combining bevacizumab with cisplatin and the HIF inhibitor topotecan (NCT0054818). The proteasome inhibitor bortezomib, which represses HIF protein expression (Befani *et al*, 2012), is being trialed in combination with everolimus in relapsed or refractory-free lymphoma (NCT00671112). Bevacizumab is also being combined with the mTOR inhibitor, MLN0128 (NCT02142803). Targeting mTOR has dual effects, as it regulates HIF expression and is an independent metabolism regulator (Shimobayashi & Hall, 2014). A completed phase I clinical trial in solid tumours combined bevacizumab with SN-38, a camptothecin analogue which reduced expression and transcriptional activity of HIF1α (Jeong *et al*, 2014a).

There are a number of clinical trials investigating targeting hypoxia or metabolism without anti-angiogenic therapy. There are 20 TH-302 hypoxia prodrug entries on clinical trials.gov. An additional study targeted HIF1α directly with EZN-2968 identifying a variety of patient tumour responses from 94% reduction in HIF1α expression to increased expression (Jeong *et al*, 2014b).

Clinical trials are investigating metformin in recurrent ovarian, fallopian tube or primary peritoneal cancer, and pancreatic cancer (NCT02050009, NCT02122185, NCT01666730, NCT01954732). The inhibitor of autophagy hydroxychloroquine is being used before surgery as a single agent in patients with primary RCC (NCT01144169). PFK-158 an inhibitor of PFKFB3 is in a phase I clinical trial (NCT02044861). CB-839 an inhibitor of glutaminase, required for glutamine conversion to glutamate, is in a number of clinical trials including in solid tumours (NCT02071862). There is a clinical trial of the MCT1 inhibitor AZD3965 in patients with advanced cancer (NCT01791595). There is also a clinical trial of a CA9 inhibitor DTP-348 (NCT02216669). It is also important to consider the effect of any metabolic targeting therapy on endothelial cell metabolism and the impact of this on angiogenesis (Goveia *et al*, 2014). Clearly, once a dose is safely selected, combination studies with anti-angiogenic drugs should be a high priority.

## Patient selection

Given the interrelatedness of vascular, hypoxic and metabolic tumour responses to anti-angiogenic therapy, it may be important to measure all three. It would be best to target normoxic tumour metabolism if the vasculature is normalised for the time frame of this response followed by targeting hypoxic tumour response when tumour perfusion is reduced. Imaging tumour perfusion and hypoxia over the treatment course would be required to find the best-fit treatment. Diffusion contrast-enhanced magnetic resonance imaging (DCE-MRI), with gadolinium contrast agent, enables non-invasive measurements of tumour perfusion and has been utilised to assess patient response to pre-operative chemotherapy with bevacizumab (De Bruyne *et al*, 2012).

Enhanced glucose uptake can be monitored by imaging fluorodeoxyglucose-PET (FDG-PET). FDG-PET is used to measure drug response (Vera *et al*, 2014). MRS analysis of cediranib treatment of glioblastoma patient tumours showed that a high n-acetylaspartate (NAA)/choline ratio was associated with increased overall survival (Kim *et al*, 2011). Additional metabolic tracers, for use in PET imaging, important in hypoxic and/or anti-angiogenic therapy response such as glutamine, would also be interesting to investigate and are in development (Schulze & Harris, 2012).

Clinical trials investigating the use of markers of hypoxia in response to anti-angiogenic therapy include using 18F-FMISO-PET to provide a hypoxic index in cerebral tumours (NCT01200134). FMISO-PET and vascular MRI scans are being utilised to explore blood flow, volume and hypoxia in bevacizumab-treated glioblastoma patients (NCT02076152). It is also important to investigate HIF response, which will have an impact on changes in gene expression patterns and therefore molecular physiology. Investigation of CA9 expression by *in vivo* imaging with radiolabeled cG250 (Stillebroer *et al*, 2013), immunohistochemistry in biopsies or measurements of hypoxia-induced proteins in the blood (Takacova *et al*, 2013) may provide HIF response information. Imaging pH in patient samples may act as a marker for susceptibility to targeting pH regulation or for samples with high metabolic rates or high levels of hypoxia. A number of strategies for imaging pH in patients have been and are being developed (Wu *et al*, 2013). Analysis of metabolites in the blood or urine of patients may also enable patient selection for targeted therapies (Lodi *et al*, 2013). Expression analysis of hypoxic gene signatures in patient baseline biopsies and biopsies during therapy would identify tumour response (Fox *et al*, 2014).

As changes in DCE-MRI occur within 1 week of anti-VEGF therapy (Ferl *et al*, 2015) and the xenografts show rapid changes, it should be a priority to examine short-term induced adaptations to help determine the most appropriate therapy for the adapted tumour, by imaging or biopsies, or peripheral blood markers.

## Future directions

It is clear that anti-angiogenic therapy, alone or with therapies targeting fast growing tumour cells, is not going to be curative in the majority of tumour patients. As with nearly all areas of cancer biology, the complexity, heterogeneity and plasticity of tumour cells and their environment were underestimated.

Given the prognostic implications of inducing the hypoxic tumour response, the utilisation of expensive and toxic drugs without selecting the correct patients can be prevented with appropriate designs for the use of precision medicine.

Targeting tumour metabolic reprogramming is a fast developing area of translational biology, and its relationship with angiogenesis and hypoxia is intertwined. It is important that the relationship between other targeting strategies and microenvironmental heterogeneity is investigated. The metabolic effects and oxygen, pH and nutritional gradients are recapitulated far better in more complex, 3-dimensional co-culture models (Thoma *et al*, 2014), which with analytical methods that enable more tumour-like *in vitro* analysis need to be more widely used. The use of pre-selection ‘window’ (between first diagnosis and tumour resection) trials for combination therapies could reduce long-term incorrect treatment strategies for patients if early response stratifying biomarkers can be developed. Furthermore, heterogeneous tumour metabolic and hypoxic responses will greatly impact the success of combination approaches and understanding fully which variations are important therapeutically and how to incorporate identification of these into treatment regimes will require further investigation.

There now exists a dichotomous future to ‘anti-angiogenic’ therapy, (perhaps better referred to as vascular adjustment therapy) focused on either increasing the effects of vascular normalisation to increase perfusion or reducing vascular perfusion and treating in combinations including hypoxia/metabolic targeting agents. Vascular normalisation has two key benefits. The first is increased delivery of chemotherapy, which has been identified to improve survival, and oxygen, which will increase the efficacy of radiotherapy. The second benefit is the reduction of hypoxic adaptation, which will reduce the negative aspects to the patient of selecting a more aggressive tumour. Reducing vascular perfusion has the advantage of the induced essentiality approaches discussed here.

Further problems remain. We cannot decipher in advance which type of vascular response patients will have. Understanding the mechanisms behind the different positive vascular responses and producing a clinically useable biomarker is a key area for progress in the paradigm of induced essentiality as one approach to personalised cancer medicine. Furthermore, there remains a subset of patients for which there will be no vascular response. Controlled clinical analysis of the effects of differing anti-angiogenics particularly given the more general tumour effects of the RTKi approaches and the impact these may have on heterogeneous subsets of patients is required.

To take advantage of the induced essentiality opportunities that arise from reduced perfusion, the field should focus on approaches that are likely to produce a kill effect rather than inhibit proliferation, as may be the case with many metabolic targeting strategies.

Utilising a hypoxia-activated prodrug such as TH-302 is an exciting approach, and the four on-going clinical trials combining this with anti-angiogenic therapies may provide strong support for the rationale. Regarding metabolic inhibitors, an example of the desired effect of apoptotic induction was identified in xenograft studies with DCA in combination with sorafenib (Shen *et al*, 2013). This may be due to the effects of DCA on multiple metabolic pathways. With this in mind, an area for possible exploitation in this combination approach is that of epigenetic modulation of gene expression patterns, enabling changes in a large number of ‘essential’ genes with a single inhibitor. Identification of epigenetic modulators of global hypoxic/metabolic gene expression patterns would facilitate more global hypoxia/metabolic targeting in combination with anti-angiogenics.

## Pending issues

- Can we better comprehend the mechanistic differences for the different vascular responses of vascular normalisation versus reduced perfusion?
- Based on this understanding can early predictors of response to anti-angiogenic therapy be identified and incorporated clinically?
- Will anti-angiogenic therapy and hypoxia/metabolic targeting combination approaches be as successful as pre-clinical data suggests?

## Abbreviations

Angiogenesis: The process of growing additional blood vessels.
Anti-angiogenic therapies: Drugs that target the tumour blood vessels and prevent the formation of new blood vessels into the tumour.
Hypoxia: Conditions of low oxygen and nutrients.
Hypoxia-inducible factor (HIF): The key regulator of the changes in gene expression that occur in conditions of low oxygen.
Hypoxic and metabolic adaptation to anti-angiogenic therapy: Changes that occur in tumour cells in response to reduced blood flow that allow them to survive in conditions of low oxygen such as in the way they make energy and the building blocks to make more cells.
Induced essentiality: Making tumour cells dependent on a pathway/protein for their ability to grow and survive by changing their microenvironment with a therapy.
Patient selection: Patients do not respond in the same way to drugs including anti-angiogenic therapy. It is important to choose how to treat patients based on how they will respond.
Tumour metabolism: Tumours change their metabolism to increase their ability to divide more rapidly by increasing the amount of cell building blocks they make and also protect them from free radical damage.
Vascular endothelial growth factor (VEGF): The key regulator of angiogenesis.
